# In Vitro and In Silico Assessments of Curcuminoids and Turmerones from *Curcuma longa* as Novel Inhibitors of *Leishmania infantum* Arginase

**DOI:** 10.3390/ph18060851

**Published:** 2025-06-06

**Authors:** Flora F. S. Spíndola, Anderson S. Pinheiro, Maria Athana Mpalantinos, Jefferson R. A. Silva, Walter S. M. F. Neto, Raissa A. Conceição, Eduarda M. Barreto, Barbara A. Abrahim-Vieira, Carlos R. Rodrigues, Alessandra M. T. Souza, Dirlei Nico, Ana Claudia F. Amaral, Andreza R. Garcia, Igor A. Rodrigues

**Affiliations:** 1 Programa de Pós-Graduação em Ciências Farmacêuticas, Faculdade de Farmácia, Universidade Federal do Rio de Janeiro, Rio de Janeiro 21941-902, RJ, Brazil; flora_fernanda@hotmail.com (F.F.S.S.); raissa.conceicao9@gmail.com (R.A.C.); babi_abrahim@hotmail.com (B.A.A.-V.); rangelfarmacia@gmail.com (C.R.R.); amtsouza2@yahoo.com.br (A.M.T.S.); 2 Departamento de Bioquímica, Instituto de Química, Universidade Federal do Rio de Janeiro, Rio de Janeiro 21941-902, RJ, Brazil; pinheiro@iq.ufrj.br; 3 Laboratório de Produtos Naturais e Derivados, Farmanguinhos Fiocruz, Manguinhos, Rio de Janeiro 21041-250, RJ, Brazil; maria.mpalantinos@gmail.com (M.A.M.); aamaral_99@yahoo.com.br (A.C.F.A.); 4 Laboratório de Cromatografia, Departamento de Química, Instituto de Ciências Exatas, Universidade Federal do Amazonas, Manaus 69077-000, AM, Brazil; jrocha01@yahoo.com.br (J.R.A.S.); wssottoo@gmail.com (W.S.M.F.N.); 5 Departamento de Fármacos e Medicamentos, Faculdade de Farmácia, Universidade Federal do Rio de Janeiro, Rio de Janeiro 21941-902, RJ, Brazil; brrduda@gmail.com; 6 Departamento de Microbiologia Geral, Instituto de Microbiologia Paulo de Góes, Universidade Federal do Rio de Janeiro, Rio de Janeiro 21941-902, RJ, Brazil; dirlei@micro.ufrj.br; 7 Departamento de Produtos Naturais e Alimentos, Faculdade de Farmácia, Universidade Federal do Rio de Janeiro, Rio de Janeiro 21941-902, RJ, Brazil

**Keywords:** antileishmanial, curcumin, turmerone, ADMET, molecular docking, enzymatic inhibition

## Abstract

**Background/Objectives**: The anti-*Leishmania* potential of *Curcuma longa* and its derivatives, such as curcuminoids, is well-established, yet their mechanisms of action remain underexplored. This study investigates the inhibitory effects of *C. longa* extracts and curcumin on *Leishmania infantum* arginase, a key enzyme in polyamine and trypanothione biosynthesis, and evaluates their antiparasitic activity. **Methods**: Extracts were prepared via rhizome successive maceration with hexane (HEXCURC), dichloromethane (DCCURC), and ethanol (ETOHCURC) and chemically characterized by a combination of chromatographic and spectrometric methods. The inhibition of recombinant *L. infantum* arginase (*Li*ARG) was assessed by urea quantification, while molecular docking explored interactions between the main compounds annotated in the extracts and the enzyme’s active site. Biological activity was tested against *L. infantum* promastigotes, intracellular amastigotes, and mammalian cells. **Results**: LC-MS and GC-MS revealed curcuminoids and turmerones as main compounds annotated in the extracts. DCCURC, HEXCURC, and curcumin showed the strongest *Li*ARG inhibition (IC_50_ = 10.04, 14.4, and 17.55 μg/mL, respectively). Docking analysis revealed that curcumin, demethoxycurcumin, and bisdemethoxycurcumin bind near the active site, with binding energies of –3.43, –4.14, and –3.99 kcal/mol, respectively. Curcumin demonstrated superior anti-promastigote activity (IC_50_ = 15.01 μg/mL) and selectivity (SI = 12.7) compared to the extracts. It also significantly reduced amastigote burden in infected macrophages (IC_50_ = 13.6 μg/mL). **Conclusions**: This is the first report demonstrating that *C. longa* extracts and curcumin inhibit *Li*ARG. These findings support curcumin’s potential as a lead compound for developing multi-target therapies against leishmaniasis, combining enzyme inhibition with direct antiparasitic effects.

## 1. Introduction

*Leishmania* spp. (Trypanosomatidae) are medically significant protozoa that cause a wide range of clinical manifestations collectively known as leishmaniasis. Infections caused by these parasites can lead to ulcerated lesions on the skin and mucous membranes, as tegumentary leishmaniasis, or they can affect internal organs, resulting in visceral leishmaniasis [1]. The treatment of choice, primarily based on pentavalent antimonials, amphotericin B, and miltefosine, depends on several factors such as the parasite species involved and the patient’s immune status. Despite the therapeutic arsenal, the drugs do not provide a sterile cure, and the disease can reemerge specially in cases of immunosuppression [2]. Additionally, current treatments face several challenges, including the emergence of resistant parasite strains, high toxicity, variable efficacy, and high rates of treatment abandonment [3,4]. In this scenario, identifying new drug candidates that target key metabolic pathways in parasites may represent a strong strategy for more efficiently combating leishmaniasis.

Arginase has emerged as a significant therapeutic target against leishmaniasis. The hydrolysis of L-arginine by arginase is the first step in the biosynthesis of polyamines, cationic molecules fundamental for transmembrane transport and parasite differentiation. Additionally, polyamines participate in the synthesis of trypanothione, which plays a protective role against reactive oxygen species (ROS) that can induce parasite death [5]. Host cells, particularly macrophages, also express the enzyme, but this is not considered an issue since arginase competes with inducible nitric oxide synthase (iNOS) for the L-arginine substrate. Inhibiting host cell arginase may therefore enhance their microbicidal activity by increasing the availability of L-arginine for iNOS [6]. Several anti-*Leishmania* agents have demonstrated inhibitory activity against *Leishmania* arginase, with flavonoids being the most extensively studied [7,8,9,10]. Additionally, cinnamic acid derivatives [10,11], chalcones [12], phenylhydrazides [13], fucogalactan [14], and benzimidazole [15] have also exhibited inhibitory effects.

*Curcuma longa* (Zingiberaceae), native to Southeast Asia, has been widely distributed across the world, particularly in tropical and subtropical regions [16]. The rhizome of *C. longa* is traditionally used as a food ingredient for its distinctive flavor and digestive properties [17]. Extracts from *C. longa* and its major components, especially curcuminoids and turmerones, have been extensively studied as bioactive agents against various illnesses, including leishmaniasis. These chemical classes have served as starting points for synthesizing new compound analogues [18,19], developing nanoformulations [20,21], exploring combination therapies [22,23], and employing photodynamic therapy [24,25], all showing great potential for disease control. Previously described mechanisms of action include apoptosis induction, the increased production of reactive oxygen species (ROS), mitochondrial dysfunction, the disruption of calcium homeostasis, the depletion of trypanothione, and enzymatic inhibition [18,20,26,27].

In the present study, we describe a novel mode of action of curcuminoids and turmerone-rich extracts from *C. longa* against *Leishmania*. The hexane, dichloromethane, and ethanol extracts and the main component curcumin inhibited *L. infantum* recombinant arginase (*Li*ARG) in addition to killing the parasite. In silico analysis revealed that other curcuminoids and turmerones present in the fractions are also capable of binding to the enzyme. These findings provide a new mode of action of *C. longa* main compounds, especially curcumin, against *Leishmania* parasites.

## 2. Results and Discussion

### 2.1. Phytochemical Profile of C. longa

The results obtained by GC-MS revealed the predominance of sesquiterpenes in the hexane (HEXCURC), dichloromethane (DCCURC), and ethanol (ETOHCURC) extracts. In the HEXCURC extract, the major compounds annotated were α-curcumene (14.3%), β-turmerone (58.8%), and α-turmerone (2.9%). β-Turmerone (23.6% and 26.6%), ar-turmerone (20.8% and 27.2%), and α-turmerone (16.0% and 14.3%) were the main constituents annotated in the DCCURC and ETOHCURC extracts, respectively. These findings align with previous results described by our group [20]. The present study also focused on the curcuminoid-rich profiles of the DCCURC and ETOHCURC extracts, as determined by LC-MS chromatographic analysis. Based on the structural characteristics of curcuminoids, these compounds contain two keto groups at positions C3 and C5, with variations in the presence of double bonds within the central chain and substituents attached to the aromatic ring. The three curcuminoids identified via LC-MS HR belong to this group and were annotated as curcumin (**1**), demethoxycurcumin (**2**), and bisdemethoxycurcumin (**3**). Their identification was based on ions detected in negative mode [M–H]⁻ at *m*/*z* 307.0949 (C_19_H_17_O_4_), 337.1055 (C_20_H_19_O_5_), and 367.0952 (C_21_H_21_O₆), respectively. The predominant compounds in DCCURC were **2**, accounting for 78%, and **1**, comprising 12%. Meanwhile, in ETOHCURC, the primary constituents were **1** (65%) and **3** (28%). These compounds differ in the number of methoxy groups: **1** contains two, **2** one, and **3** none.

Notably, the extracts demonstrated a solvent-dependent distribution of stereoisomers within the turmerone group (Supplementary Material, Figure S1). A similar trend was observed for curcuminoids. Consequently, the extraction methodology proved effective in partitioning the sesquiterpenes and curcuminoids across the different extracts. This distribution significantly influenced the biological activities described below, underscoring the importance of extraction methods in determining the bioactivity of plant-based preparations.

### 2.2. LiARG Inhibition In Vitro

The inhibitory activity of *C. longa* extracts and **1** against purified recombinant *Li*ARG was assessed across a range of concentrations (Supplementary Material, Figure S2). Among the samples tested, DCCURC showed the lowest IC_50_ value (10.04 ± 1.5 μg/mL), followed by HEXCURC (14.4 ± 2.4 μg/mL), **1** (17.55 ± 0.4 μg/mL) and ETOHCURC (52.64 ± 0.3 μg/mL) (Table 1). Statistically, the activity of DCCURC, HEXCURC, and **1** was similar, while ETOHCURC was less active. The similar inhibitory activity of HEXCURC and DCCURC to that of **1** suggests that sesquiterpenes turmerones, abundant in HEXCURC, and 2, present in DCCURC, may also contribute to LiARG inhibition as part of their mechanism of action.

There are few reports regarding curcuminoids’ effect on arginase. The treatment with **1** was able to reduce mouse arginase activity by 73% in a colorectal cancer model [28]. There is also a lack of **1** inhibitory activity on *Leishmania* arginase. Its antileishmanial effects have previously been associated with the induction of apoptosis [29] and the reduction of plasma membrane fluidity [30].

Similarly, to the best of our knowledge, there are no reports on the inhibitory activity of sesquiterpene compounds, such as turmerone, on *Leishmania* arginase. A previous report demonstrated that the sesquiterpene alismoxide, isolared from *Curcuma comosa*, showed inhibitory activity against human arginase I [31]. The mechanisms of action attributed to turmerone against *Leishmania* include morphological and ultrastructural alterations [20] and an increase in metacaspase expression, which acts in cell death pathways [32]. In macrophages, turmerones have antioxidant and immunomodulatory activities, such as NO inhibition [33], and a significant increase in gene expression for IL-12 p40, 1L-1β, and iNOS, indicating a TH1 response, important for leishmaniasis control [32].

### 2.3. LiARG In Silico Binding Mode Analysis

**Figure 1:**
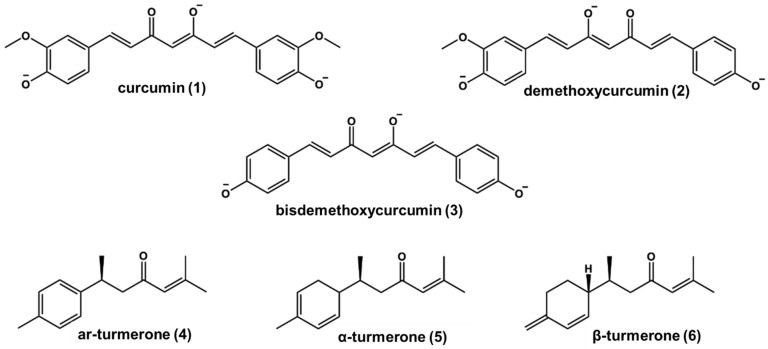
Chemical structures of curcumin (**1**), demethoxycurcumin (**2**), bisdemethoxycurcumin (**3**), and turmerones (**4**–**6**) in alkaline media.

Molecular docking studies were performed to determine the binding modes of curcuminoids and turmerones **1**–**6** with *Li*ARG. After the validation of the docking protocol (Supplementary Material, Figure S3), the protein and the main compounds were considered in the ionized state at pH 9.5 (Figure 1) according to the literature and experimental studies [34]. The overall analysis of the complexes obtained between *Li*ARG and curcuminoids **1–3** indicated that these ligands were positioned between protein loops at the entry of the active site and showed similar binding mode values of −3.43 kcal/mol, −4.14 kcal/mol, and −3.99 kcal/mol, respectively.

**Figure 2:**
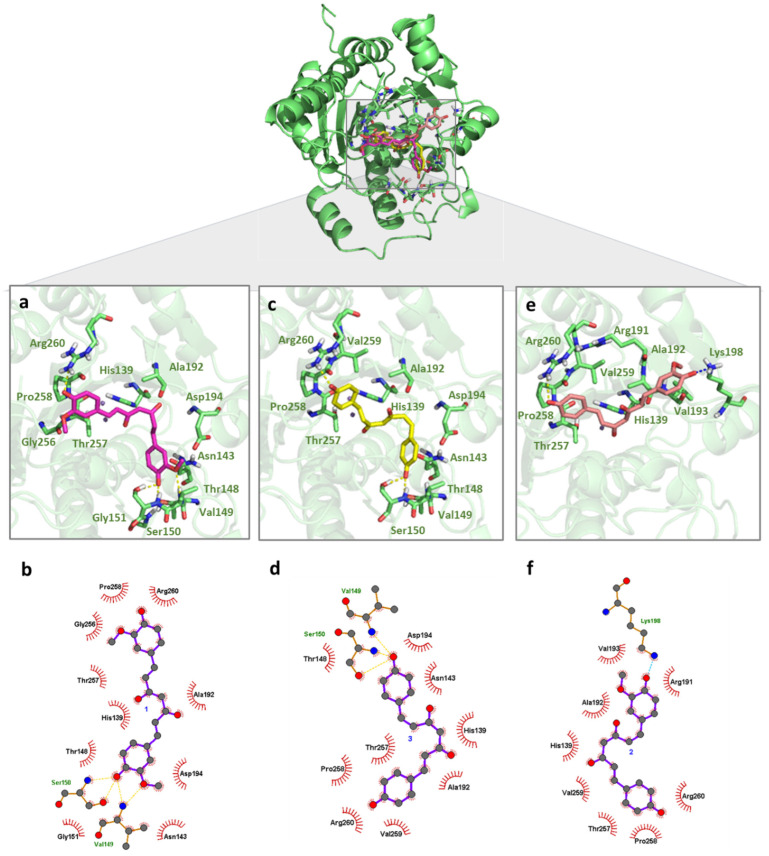
Binding mode of curcuminoids with *Li*ARG obtained by molecular docking. The superposition of the final docking pose of each inhibitor docked into the active site of *Li*ARG is shown in the center. The three-dimensional model of *Li*ARG is displayed in cartoon representation and colored green. The molecular structures of (**a**) curcumin (**1**) (pink), (**c**) bisdemethoxycurcumin (**3**) (yellow), and (**e**) demethoxycurcumin (**2**) (light pink) are shown in stick representation. Residues that participate in interactions are colored in light green. The 2D diagrams show the interactions between (**b**) **1**, (**d**) **3,** and (**f**) **2**. Hydrogen bonds and ionic interactions are depicted as yellow and blue dashed lines, respectively.

Curcuminoids have similar chemical structures except for the number of methoxy groups on each of the two phenyl moieties, which affect their binding modes. Binding mode analysis of *Li*ARG:**1** and *Li*ARG:**3** complexes indicated that both inhibitors adopted similar orientations and interactions within the binding site. Indeed, their phenolate moieties play an important role in the complex formation. One phenolate may interact with Ser150 through hydrogen bonds with its hydroxyl (OH—O- distances of 2.7 Å) and main chain (NH—O distances of 2.9 and 2.6 Å, respectively). Moreover, the second phenolate interacts with Arg260 through hydrogen bonds with its side chain (NH—O- distances of 2.6 and 2.8 Å, respectively). Due to the presence of the methoxyl group, **1** establishes an additional hydrogen bond as acceptor with Val149 (NH—O distance of 3.2 Å). Van der Waals interactions with His139, Asn143, Thr148, Gly151, Ala192, Asp194, Thr257, and Pro285 residues also contributed to the formation of both complexes. Gly151 and Gly256 residues also participate in *Li*ARG:**1** and Val259 residues and in *Li*ARG:**3** complexes (Figure 2a,b).

For the complex *Li*ARG:**2**, the monosubstituted aromatic ring maintained the hydrogen bond interaction with Arg260 (NH—O distance of 3.0 Å), while the disubstituted aromatic ring is oriented to the Lys198 residue for an ionic interaction through its deprotonated hydroxyl (NH—O distance of 2.4 Å). In the same way as the two earlier complexes, van der Waals interactions with His139, Ala192, Thr257, Pro258, and Val259, in addition to Arg191 and Val193, also contributed to the formation of *Li*ARG:**2** (Figure 2c). Possibly due to the long chain spacer connecting the phenolate extremities, the curcuminoids interact with external loops of the *Li*ARG impacted in the estimated binding energy. Even so, residues as His139 and Pro258 that participated in the interactions with curcuminoids were also found before by our group to interact with rosmarinic acid [10]. Additionally, a hydrogen bond with Arg260 was among the interactions displayed by the chalcone derivative identified as effective against promastigote and amastigote forms of *L. infantum* [12].

**Figure 3:**
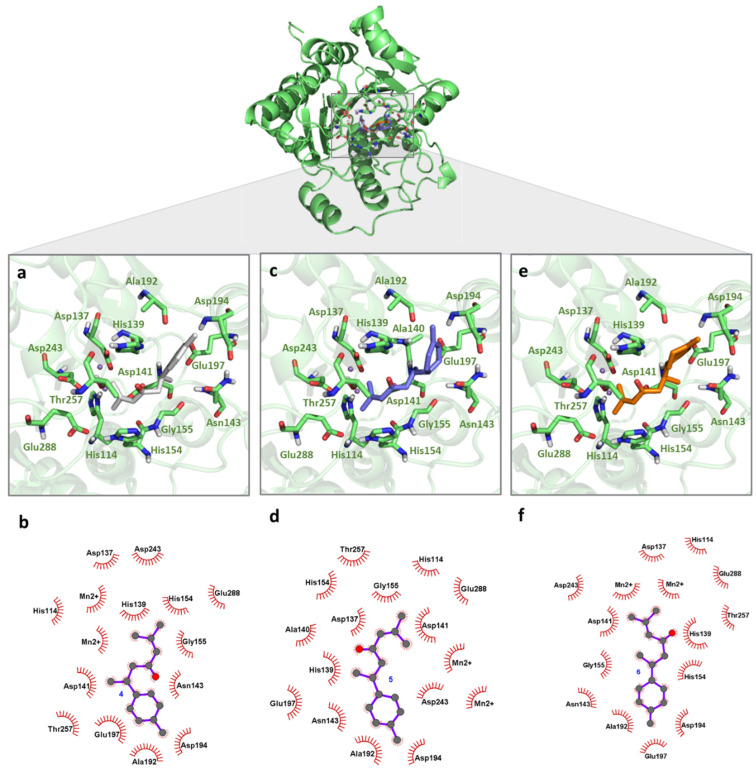
Binding mode of turmerones with *Li*ARG obtained by molecular docking. The superposition of the final docking pose of each inhibitor docked into the active site of *Li*ARG is shown in the center. The three-dimensional model of *Li*ARG is displayed in cartoon representation and colored green. The molecular structures of (**a**) ar-turmerone (**4**) (grey), (**c**) α-turmerone (**5**) (purple), and (**e**) β-turmerone (**6**) (orange) are shown in stick representation. Residues that participate in interactions are colored in light green. The 2D diagrams show the interactions between (**b**) **4**, (**d**) **5,** and (**f**) **6**.

Regarding the turmerone complexes **4**–**6**, all ligands assumed similar orientations among them and deeper positions in the *Li*ARG active site when compared to curcuminoids. Here, van der Waals interactions contributed to the lower estimated binding energy values (**4**, −5.90 kcal/mol, **5**, −6.20 kcal/mol, **6**, −6.22 kcal/mol), and no electrostatic interactions were observed. Despite the modification of the oxidative pattern of the ring moiety in their chemical structure, no significant changes in binding mode were observed. The α,β-unsaturated carbonyl moiety was placed between His139 and His154 while the alkene extremity was placed among Asp137, Asp141, Asp243, Glu288, and Mn2+ ions (Figure 3a–c). These residues are conserved among all arginases and have been described as key residues for potential inhibitors binding with other *Leishmania* species [15,35,36,37,38,39]. His114, Ala140, Ile142, Asn143, Gly155, Ala192, Asp194, Glu197, and Thr257 residues also participate in complexes’ formation, increasing the affinity of turmerones to the enzyme. These results indicate the potential of turmerones **4**–**6** to act as inhibitors against arginase of *L. infantum*.

### 2.4. In Silico ADMET Profile

The use of in silico methods to provide data on the pharmacokinetics and toxicity of hit compounds is an attractive and cost-effective alternative to support decision making in drug development programs for leishmaniasis. Therefore, the curcuminoids and turmerone (**1**–**6**) were submitted to pharmacokinetic and toxicological profile evaluations using Quantitative Structure–Activity Relationship (QSAR)-based models implemented in VEGA v.1.2.3 and ADMET Predictor ™ v11.0 (Simulation Plus, Lancaster, CA, USA) softwares. The pharmacokinetic parameters evaluated included oral bioavailability, as determined by the Lipinski rule, the percentage of unbound substance in the plasma, and the likelihood of being a P-glycoprotein (Pgp) substrate (Table 2).

The results presented herein suggested that these substances exhibit good oral bioavailability after passive oral absorption based on physicochemical properties and low fraction unbound to plasma protein, ranging between 6.2 and 12.1% freedom in the plasma. These are the fractions that will be able to permeate cell membranes, interact with the target protein, and be metabolized. Although this feature may directly impact pharmacodynamics, it may contribute to a longer half-life for these substances [40]. Studies have shown that **1** stability is improved by the main plasma proteins such as human serum albumin (HSA) and fibrinogen [41]. Molecular docking analyses demonstrated that **1** interacts with HSA through the carbonyl oxygen and hydroxyl oxygen of one phenolate [42], these functional groups are also present in the structures of **2** and **3**. HSA is a positively charged protein and has hydrophobic pockets, and then acidic and neutral drugs, respectively, can selectively bind to the protein, resulting in a low unbound fraction in the plasma [43].

Considering the P-glycoprotein interaction, all the compounds, with the exception of **4**, were substrates and inhibitors of the transporter. Chearwae and co-workers’ (2004) studies suggested that the curcuminoids **1**–**3** bind to substrate-binding sites on Pgp and inhibit the transporter but are not substrates [44]. However, the study by Yue and co-workers demonstrated that in the presence of turmerones **4** and **5**, **1** efflux is reduced. Additionally, the authors showed that only **5** is a Pgp inhibitor since it was able to affect the uptake of rhodamine-123 and digoxin, known Pgp substrates. The results indicated that **1** uptake was increased because of Pgp inhibition by **5** [45]. No related study was found for **6**. Then, all together these results contribute to confirm the in silico prediction and solve the influence of **6** on Pgp activity.

### 2.5. Cytotoxic and Hemolytic Effects of C. longa Extracts and Curcumin

After 48 h of treatment, a dose-dependent effect was observed on cell death for both RAW 264.7 and VERO cell lines (Supplementary Material, Figure S4). The 50% cytotoxic concentration (CC_50_) values ranged from 70.16 to 47.87 μg/mL for RAW 264.7 cells and from 89.3 to 58 μg/mL for VERO cells (Table 3). These values were significantly higher than the CC_50_ values of the reference drug control, PAT (potassium antimonyl(III) tartrate hydrate), as a source of trivalent antimony (Sb(III)), which were 30.8 ± 0.73 μg/mL for RAW 264.7 cells and 23.9 ± 1.5 μg/mL for VERO cells, respectively.

The curcuminoid-rich fraction showed approximately ten times higher cytotoxicity toward VERO cells compared to compound **3**, while the ethanolic extract was the least cytotoxic, with a CC_50_ of 525 µg/mL [46]. Scientific evidence indicates that curcumin (**1**), at non-toxic doses, exerts hepatoprotective effects [47]. Moreover, **1** has demonstrated the potential to prevent and treat acute kidney injury induced by drugs such as amphotericin B through a modulation of the immune response, a reduction of inflammatory markers, decreased apoptosis, and improved mitochondrial dynamics [48]. Clinical studies have also confirmed that the oral administration of standardized turmeric and curcumin extracts is safe for human use [49].

Cytotoxicity was also evaluated through the hemolytic potential of *C. longa* extracts against sheep red blood cells (RBC). The results demonstrated that there is a tendency for hemolysis to occur as the concentration of the extracts increases (Supplementary Material, Figure S4). Amin and Dannenfelser (2006) proposed a notable guide for classifying the hemolytic potential of pharmaceutical excipients, marking those with values exceeding 25% hemolysis as potentially hemolytic [50]. For *C. longa* extracts, hemolysis did not exceed 25% up to 100 μg/mL. The 50% hemolytic concentrations (CH_50_) were 119.65 ± 3.4 (HEXCURC), 101.85 ± 9.6 (DCCURC), and 144.15 ± 13.35 (ETOHCURC) μg/mL (Table 3). PAT did not show hemolytic potential at the highest concentration tested (100 μg/mL).

### 2.6. Antileishmanial Activity

Initially, a screening of the activity of *C. longa* extracts and **1** was performed against *L. amazonensis* and *L. infantum* promastigote forms, showing a dose–response effect (Supplementary Material, Figure S5). The IC_50_ for *L. amazonensis* ranged from 5.5 to 102.3 μg/mL, while for *L. infantum* it ranged from 15.5 to 74.7 μg/mL (Table 4). Interestingly, a previous study described that a mix of curcuminoids exhibits better biological activity than curcumin alone [51]. Here, **1** exhibited better activity than the extracts against both *Leishmania* species, achieving the highest selectivity index (SI) values: 12.7 for *L. amazonensis*, which was higher than that of the reference drug control, PAT, and 4.5 for *L. infantum.* In contrast, the extracts displayed SI values that indicate their important cytotoxicity in the host macrophage lineage tested here (Table 4).

The turmerone-rich extract, HEXCURC, had the lowest selectivity index (SI) for both *Leishmania* species, being more toxic to host cells than to the parasite. Teles et al. (2019) reported the presence of 55.43% turmerone and 12.02% β-turmerone in the essential oil of *C. longa*. This essential oil showed no cytotoxicity to BALB/c peritoneal macrophages at the highest tested concentration (1000 μg/mL) and exhibited an IC_50_ of 63.3 ± 1.369 µg/mL against intracellular *L. amazonensis* amastigotes [33]. Additionally, ar-turmerone alone demonstrated low cytotoxicity against J774.A.1 macrophages, with a CC_50_ of 720.06 μg/mL and a SI of 21.1 for *L. major* amastigotes [32]. Therefore, further studies are needed to assess the cytotoxicity of turmerones against RAW 264.7 and VERO cells, as the toxicity observed in this study may be attributed to other components of the HEXCURC extract. Despite the potential hepatotoxicity for **5** and **6**, the in silico toxicological parameters of turmerones were satisfactory. In addition, all compounds were approved in the Ames test, as well as carcinogenicity, acute toxicity, and cardiotoxicity assessments.

There are limited data on the effect of **2**, the predominant curcuminoid in the DCCURC extract, against *Leishmania* spp. In the study by Rasmussen et al. (2000), demethoxycurcumin exhibited an IC_50_ of 14.1 ± 4.8 µg/mL against *L. major* promastigotes, showing greater potency than bisdemethoxycurcumin (IC_50_ = 21.5 ± 6.9 µg/mL) but lower potency than curcumin (IC_50_ = 7.8 ± 3.3 µg/mL) [52].

The genus *Leishmania* includes more than 20 pathogenic species [1] and numerous strains with varying sensitivity to antileishmanial agents, which could partly explain the variability in treatment effectiveness [53]. Both species tested in this study were sensitive to compound **1**. Previous studies have also demonstrated the activity of this compound against promastigotes of *L. amazonensis* [54] and *L. infantum* [55]. For intracellular amastigotes of *L. amazonensis*, photodynamic therapy using nanoparticle-formulated curcumin reduced parasite viability after 24 h of treatment [25]. Intriguingly, despite several reports of curcumin’s antileishmanial activity, there is a lack of its activity against intracellular amastigotes of *L. infantum*, the clinically relevant biological form of the parasite.

**Figure 4:**
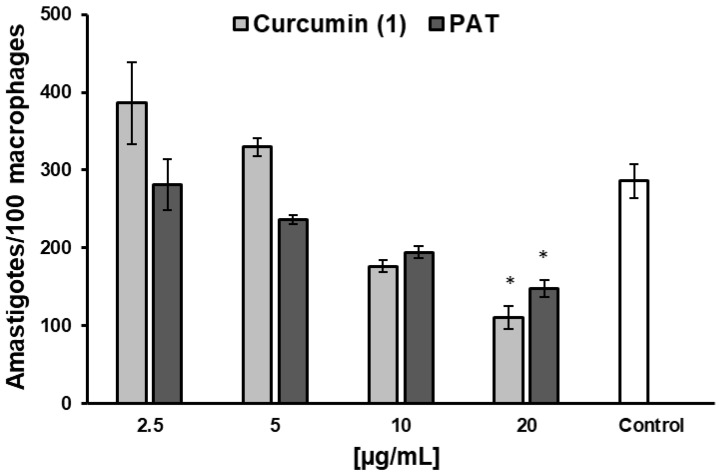
Effect of curcumin and PAT in intracellular amastigotes amounts after 48 h of treatment in *L. infantum*-infected RAW 264.7 cells. Data are expressed as the mean of two independent experiments, each performed in duplicate. Statistical analysis was conducted using One-Way ANOVA followed by Tukey’s post-test, comparing each treatment with untreated cells (control), where (*) = *p* < 0.05.

Based on promising results, **1** activity was evaluated against intracellular *L. infantum* amastigotes, resulting in an IC_50_ of 13.6 ± 0.7 μg/mL and an IS of 5.13, with a dose-dependent effect. The reference drug control PAT showed an IC_50_ of 18.9 ± 4.06 and an IS of 1.63 (Supplementary Material, Figure S6). Both treatments significantly reduced the number of amastigotes per 100 infected macrophages at a concentration of 20 μg/mL (Figure 4).

### 2.7. Effect of Curcumin on NO Production of L. infantum-Infected Macrophages

**Figure 5:**
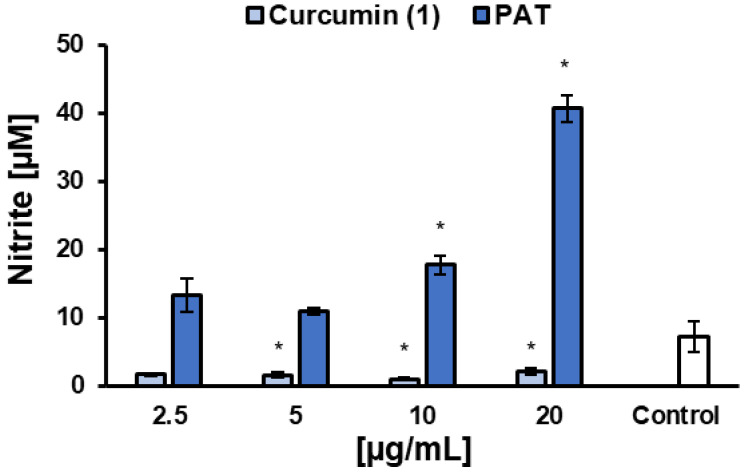
Effects of curcumin (**1**) and the reference drug control PAT on nitric oxide (NO) production by RAW 264.7 macrophages infected with *L. infantum*. Data are expressed as the mean of two independent experiments, each performed in duplicate. Statistical analysis was conducted using One-Way ANOVA followed by Tukey’s post-test, comparing each treatment with untreated cells (control), where (*) = *p* < 0.05.

NO levels were determined after 48 h of treatment with **1** in *L. infantum*-infected macrophages. The results showed that **1** reduced NO levels at concentrations starting from 5 µg/mL, compared to the untreated control, while PAT increased NO levels at 10 and 20 µg/mL (Figure 5). One of the main microbicidal mechanisms of phagocytes infected by *Leishmania* spp. is the increased production of NO by iNOS, using L-arginine as substrates [56]. The overexpression of iNOS is induced by IFN-γ as part of a Th1-type host immune response. In this study, **1** showed similar IC_50_ values (*p* = 0.05824, *t* test) against *L. infantum* promastigotes (15.5 ± 1.7 µg/mL, ~42 µM) and intracellular amastigotes (13.6 ± 0.7 µg/mL, ~36.9 µM), suggesting that its effect is likely independent of nitrosative stress and may involve molecular targets shared by both parasite forms. Interestingly, the reduction in NO production did not impair the anti-*L. infantum* activity of **1**. However, to confirm a direct action of **1** on the parasite, independent of microbicidal stimulation by the host cell, other macrophage microbicidal mechanisms must also be ruled out, such as the generation of reactive oxygen species (ROS), the myeloperoxidase system, the enhancement of lysosomal enzyme activity [57], and the activation of nuclear factor erythroid 2-related factor 2 (Nrf2) [58].

Curcumin is well known for its anti-inflammatory and antioxidant properties [59]. However, some studies suggest that agents with such properties may impair the host immune response and potentially exacerbate infection. Adapala and Chan (2008) demonstrated that the long-term oral consumption of curcumin, even at low doses, reduced the gene expression of Th1 immune response markers (iNOS, IFN-γ, and TNF-α) in organs infected with *L. donovani,* compared to untreated infected controls [60]. In the study by Chan et al. (2005), curcumin (15 µM) did not inhibit the growth of *L. major* promastigotes or promastigotes and axenic amastigotes of *L. donovani*. Moreover, curcumin reverted the inhibition parasite effects of nitric oxide-generating agents, peroxynitrite, and SIN-1 chloride [61]. Conversely, Das et al. (2008) reported that curcumin had an IC_50_ of 25 µM against *L. donovani* promastigotes and, at 20 µM, reduced intracellular amastigote load by 85% within 24 h of treatment. Additionally, curcumin increased ROS levels and lipid peroxidation in *L. donovani* promastigotes [26]. Similarly, Alinejad et al. (2022) observed elevated ROS levels following curcumin treatment in an in vitro *L. major* infection model, accompanied by an increased gene expression of IFN-γ, TNF-α, and iNOS [62]. In in vivo models, the topical administration of 1.5 mg/day of curcumin in hamsters infected with *L. braziliensis* resulted in an 83% therapeutic response [63]. Moreover, topical and intralesional treatment with curcumin nanoemulsion significantly reduced the parasite burden in *L. major*-infected mice after four weeks of treatment [64].

The variability in results reported in the literature on the antileishmanial effects of curcumin may be attributed to several factors, such as the varying sensitivity among pathogenic species of the *Leishmania* genus [53], differences in tested concentrations, treatment duration, parasite load, and the solvent of choice to solubilize curcumin, which has included DMSO [26,63], sodium hydroxide solutions or acetone [60], or in nanoformulations [64].

The results presented here highlight curcumin as a potential candidate to expand the therapeutic arsenal against visceral leishmaniasis caused by *L. infantum.* The investigation of the mechanisms of action of leishmanicidal agents is important for developing new multi-target strategies against leishmaniasis, aiming to overcome the current challenges of conventional treatments. In addition to the favorable results with **1**, the inhibition of *Li*ARG by the turmerone-rich extract (HEXCURC) highlights the inhibitory potential of these sesquiterpenes on the enzyme, suggesting a possible antileishmanial mechanism of action. This is supported by in silico molecular docking assays, which showed that all major compounds from *C. longa* formed favorable complexes with *Li*ARG, with turmerones displaying a particularly advantageous orientation within the enzyme’s active site. These findings underscore the promising potential of sesquiterpenes in targeting *Leishmania* spp. arginase.

## 3. Materials and Methods

### 3.1. Chemicals, Reagents, and Culture Media

Curcumin (**1**) (CAS number 458-37-7) was obtained from Cayman Chemical (Ann Arbor, MI, USA). Resazurin, thiazolyl blue tetrazolium bromide (MTT), propidium iodide (PI), potassium antimony(III) tartrate hydrate (PAT), and penicillin–streptomycin solution (5000 units penicillin and 5 mg streptomycin/mL) were purchased from Sigma–Aldrich (St. Louis, MO, USA). Fetal bovine serum (FBS) was purchased from LGC Biotecnologia (Cotia, SP, Brazil). The Schneider’s Insect Medium (SIM), Dulbecco’s Modified Eagle Medium (DMEM), and Grace’s Insect Medium (GIM) were purchased from Sigma–Aldrich (St. Louis, MO, USA). Dimethylsulfoxide (DMSO) was purchased from Dinâmica (Indaiatuba, SP, Brazil). All other reagents were analytical grade.

### 3.2. Plant Material and Extraction Procedures

Fresh *C. longa* rhizomes were purchased from a local market and authenticated by comparison with a voucher specimen (No. 224169) deposited at the INPA Herbarium (Herbarium of the National Institute of Amazonian Research, Manaus, AM, Brazil). The rhizomes (35 g) were crushed and subjected to sequential solid–liquid extraction using solvents of increasing polarity. Initially, the material was macerated in hexane (1 × 90 mL) under agitation and protected from light. After 5 days, the solvent was replaced, and the extraction continued. The resulting liquid phase was evaporated under reduced pressure to obtain the hexane extract (HEXCURC). The solid plant residue remaining after hexane extraction was then sequentially extracted using dichloromethane (3 × 90 mL), followed by ethanol (3 × 90 mL), under the same conditions. The combined liquid phases from each solvent were evaporated under reduced pressure, yielding the dichloromethane extract (DCCURC) and the ethanol extract (ETOHCURC), respectively. The extraction yields were calculated as the ratio between the dry extract mass and the initial plant mass (% w/w), resulting in 0.6% for HEXCURC, 2.8% for DCCURC, and 3.9% for ETOHCURC.

### 3.3. Volatile Organic Compounds

#### 3.3.1. Sample Preparation and Extraction SPME

HEXCURC, DCCURC, and ETOHCURC fractions were weighed (10 mg) and submitted to headspace—solid phase microextraction (HS-SPME). Each sample was added to 0.75 g of NaCl and 5 mL of Milli-Q water in a 20 mL vial. The flask was sealed with cap aluminum septum and placed in a water bath at 40 °C, under magnetic stirring. After the equilibration time (10 min), a Supelco SPME device with a fiber coated with 65 µm polydimethylsiloxane/divinylbenzene (PDMS/DVB) (Bellefonte, PA, USA) was inserted into the vial containing the sample. The fiber was exposed to the headspace for 40 min at 40 °C. Once sampling was finished, the fiber was withdrawn into the needle and transferred to the injection port of the gas chromatograph/mass spectrometer (GC–MS) system for 4 min at 260 °C to desorb the analytes in splitless mode.

#### 3.3.2. Volatile Compounds Analysis by GC–MS

GC–MS analyses were performed using a GC-2010 plus gas chromatograph (Shimadzu Corporation, Kyoto, Japan) interfaced with a QP-2010 Mass Selective Detector (ionization voltage 70 eV), equipped with a nonpolar DB-5MS capillary column (30 m × 0.25 mm, film thickness 0.25 μm), using helium as carrier gas (1.0 mL/min). The oven temperature was programmed from 50 °C to 260 °C, at 7 °C/min, then isothermal at 260 °C for 5 min, using H_2_ as the carrier gas (1.0 mL/min). Injector and detector temperatures were 250 °C. Linear velocity (ū) was 14 cm/s. MS interface temperature: 280 °C; mass range: 40–700 Daltons; scan speed: 150 u/s; interval: 0.50 s (2 Hz). All analyses were performed in triplicate. The identification of volatile constituents was performed by a comparison of their retention indices and mass spectra with those reported in the literature or presented in the Wiley data system library of the GC-MS equipment.

### 3.4. Identification of the Compounds by LC-QTOF-ESI-HRMS

Experimental conditions were as follows: the mobile phase consisted of (A) H_2_O with 0.1% formic acid and (B) CH₃CN. A Kinetex column (150 × 4.6 mm, 5 µm) was used, with a gradient profile as follows: 0 min/20% B, 10 min/32% B, 12 min/40% B, 14 min/52% B, 20 min/52% B, followed by a return to 20% B after 8 min. The column temperature was maintained at 30 °C, with a backpressure of 160 bar. The samples (DCCURC and ETOHCURC) were injected (5 µL), and the flow rate was set at 3 mL/min, with a split flow rate of 0.3 mL/min. Detection was performed in scan mode using electrospray ionization (ESI) in negative mode. A Q-TOF Compact Bruker mass spectrometer was used, operating in a mass range of 100 to 1000 Daltons. Sodium formate was employed as the calibrant. The ESI source parameters were as follows: nebulizer gas (nitrogen) at 5.5 bar, dry gas (nitrogen) at 12 L/min and 220 °C, capillary voltage of 4.5 kV, and QTOF/HRMS/MS. Peak data were compared with literature references and database entries for compound identification.

### 3.5. LiARG Inhibition Assay

The expression and purification of recombinant arginase from *L. infantum* (*Li*ARG) were previously detailed by our group [10]. *Li*ARG inhibition was performed by incubating different concentrations (4.7 to 100 μg/mL) of the samples in the enzymatic reaction solution: 50 mM CHES buffer (pH 9.5) and 50 mM L-arginine. The enzymatic reaction was initiated by adding *Li*ARG (10 μg/mL) and allowed to proceed for 5 min at 37 °C before being stopped on ice. Subsequently, enzymatic activity was assessed by measuring the conversion of L-arginine to urea using a commercial kit (Labtest UREA CE kit^®^, Lagoa Santa, MG, Brazil). The amount of urea produced in each reaction was determined spectrophotometrically at 600 nm, following its hydrolysis into ammonia and conversion into indophenol blue. Quercetin was used as a reference arginase inhibitor [7], and reactions without extracts or **1** served as positive controls for *Li*ARG activity. From that, the IC_50_ was determined by regression analysis.

### 3.6. Molecular Docking for Binding Mode Analysis

The molecular docking technique was employed to investigate the binding modes of the *C. longa* major components into the active site of *Li*ARG. The AutoDock 4.0 software (Scripps Research, La Jolla, CA, USA) [65] was used on a Windows-based PC for molecular docking. To validate the docking process, re-docking was performed. Since the structure of *Li*ARG complexed with an inhibitor has not been experimentally resolved, the *Leishmania mexicana* arginase (LmARG) complexed with the inhibitor 2-(S)-amino-6-boronohexanoic acid (PDB code 4IU0) [37] was selected. LmARG shares 95.18% identity with *Li*ARG, including key amino acids in the active site crucial for ligand recognition, making it highly compatible for this analysis.

#### 3.6.1. Preparation of Structure

A three-dimensional model of *Li*ARG was used for the [10,12] The Protein Prepare server was employed to protonate the titratable residues at pH 9.5 [66]. Using PyMol (The PyMOL Molecular Graphics System, Version 1.7.5.0, Schrödinger, LLC, San Francisco, CA, USA), the co-crystallized ligand and water molecules were removed. The protein was treated as rigid, nonpolar hydrogen atoms were merged, and Gasteiger charges were assigned by default using AutoDockTools (ADT). The 3D structures of the compounds were optimized and minimized with the MMFF94 force field and conjugate gradient algorithm in Avogadro 1.2.0 software (Avogadro development team, USA) [67].

#### 3.6.2. Grid Parameters

The box size for the ligands docking was defined using AutoGrid version 4, and centered on the ligand (−9.328; −22.064; 8.027). The grid map dimensions were 50 × 50 × 50 points with 0.375 A spacing. Molecular docking calculations were conducted in the AutoDock 4.0 software, and the docking files were prepared using AutoDockTools1.5.7 software (Scropps Research, La Jolla, CA, USA) [65].

#### 3.6.3. Docking Parameters and Analysis

Docking studies were carried out using the Lamarckian Genetic Algorithm (LGA). LGA default parameters such as initial population (150), number of energy assessments (2500000), mutation rate (0.02), crossover rate (0.8), and elitism (1) were kept, while the number of GA runs was modified to 50, as defined by the validation step. The results of the lowest RMSD (Å) (re-docking) and of the most favorable free energies binding in the most populated clusters (docking) were selected as possible structures of the resultant complexes. Results were analyzed using the PyMOL program (The PyMOL Molecular Graphics System, Version 1.7.5.0, Schrödinger, LLC, San Francisco, CA, USA).

### 3.7. Computational ADMET Analysis

The compounds were evaluated for pharmacokinetics and toxicity using in silico QSAR-based models implemented in VEGA HUB.1.2.3 software (Laboratory of Environmental Chemistry and Toxicology, Milan, Italy) “https://www.vegahub.eu/ (accessed on 8 January 2024)” [68] and ADMET Predictor™ version 11.0 (SimulationsPlus, Inc., Lancaster, CA, USA). For the pharmacokinetic profile, the number of violations of Lipinski’s rule were counted, and the percentage of the drug expected to remain unbound in human plasma, as well as the likelihood of P-glycoprotein interaction as a substrate or inhibitor, were assessed using ADMET Predictor™ models. For the toxicity profile, mutagenicity (based on the Ames test) and carcinogenicity in rats were analyzed using VEGA CONSENSUS v1.0.4 and CAESAR v2.1.10 models, respectively. Acute toxicity in rats, along with cardiotoxicity and hepatotoxicity in humans, were evaluated using ADMET Predictor™ models, as previously described [12].

### 3.8. Cytotoxicity Assay

RAW 264.7 (murine macrophages) and VERO (normal African green monkey kidney epithelial cells) lines were obtained from Banco de Células do Rio de Janeiro (BCRJ, RJ, Brazil) under the codes 0212 and 0245, respectively. Both were cultured in complete DMEM medium (10% FBS, 100 U/mL penicillin, 100 µg/mL streptomycin, and 3 µg/mL Fungizone^®^) at 37 °C and 5% CO_2_ atmosphere. After reaching subconfluence, the cells were washed twice with cold phosphate saline buffer (PBS, pH 7.2), detached by trypsinization, and seeded into 96-well microplates (10^5^ cells/100 μL). After an incubation period of 24 h, the extracts, **1**, or PAT were added at final concentrations ranging from 6.2 to 200 μg/mL. The cells were further incubated under the same conditions for another 48 h. Drug-free cells cultured in DMEM served as positive controls of viability. The concentration of the solvent (DMSO) was normalized in all wells (1%) of treatment and control. Cell viability was assessed using a colorimetric assay based on the reduction of tetrazolium salt [69]. The 50% cytotoxic concentration (CC_50_) was determined by regression analysis of the generated dose–response curves. In addition, the selectivity indexes (SI) for the extracts and **1** were calculated as the ratio of CC_50_ of RAW 264.7 to IC_50_ of parasites.

### 3.9. Hemolysis Assay

Red blood cells (RBCs) from *Ovis aries* (ethical approval 061/22-CEUA/UFRJ) were used to assess the hemolytic activity of the extracts and **1**. Blood samples were washed four times with cold PBS to remove plasma, and a 4% (*v*/*v*) RBC suspension was prepared in the same buffer. In 96-well microplates, 20 µL of each drug was serially diluted and mixed with 80 µL of the RBC suspension, resulting in final drug concentrations ranging from 12.5 to 200 μg/mL. After a 1 h incubation at 37 °C, the reaction was stopped by adding 200 µL of PBS. A positive hemolysis control was performed by adding 200 µL of distilled water to untreated cells. The microplates were then centrifuged (2000 rpm/5 min), and 200 µL of the supernatants were transferred to new 96-well microplates for spectrophotometric analysis at 540 nm (SpectraMax M2, Molecular Devices, CA, USA). The percentage of hemolysis for each treatment was calculated relative to the hemolysis control (100%), and the 50% hemolytic concentration (HC_50_) was determined through regression analysis of the dose–response curves.

### 3.10. In Vitro Anti-Leishmania spp. Activity

Promastigote forms of *Leishmania infantum* (MHOM/BR/1974/PP75) and *Leishmania amazonensis* (IFLA/BR/1967/PH8) were cultured in complete Schneider’s Insect Medium (SIM, 10% FBS). Antileishmanial assays were performed in 96-well microplates at 26 °C, where 10⁷ promastigotes/mL were exposed to varying concentrations (6.25 to 100 µg/mL) of the extracts, **1,** or PAT (reference drug control). Drug-free parasite cultures served as a positive control of viability. The concentration of the solvent (DMSO) was normalized in all wells (1%) of treatment and control. After 24 h of promastigote incubation, 100 μM resazurin was added, and the cells were cultured for an additional 24 h [70]. At 48 h of the treatment, parasites were assessed fluorometrically (555 nm excitation and 585 nm emission) for viability using a microplate reader Spectra Max M2 (Molecular Devices, CA, USA). Dose–response curves were then generated, and the half-maximal inhibitory concentrations (IC_50_) for each treatment were determined through regression analysis.

### 3.11. In Vitro Infection and Inhibition of Intracellular Amastigote Activity

RAW 264.7 macrophages in complete DMEM medium (10% FBS, 100 U/mL penicillin, 100 µg/mL streptomycin) were seeded in 24-well plates (2 × 10^5^ cells/well) containing round coverslips. After adhesion, the cells were incubated for 24 h in the presence of *L. infantum* promastigotes at a ratio of 10 parasites per macrophage at 35 °C in a 5% CO_2_ atmosphere. After the interaction period, the wells were washed with PBS to remove non-internalized promastigotes, and the infected macrophages were incubated for 24 h under the same conditions. Subsequently, infected macrophages were treated with different concentrations of **1** and PAT. Untreated infected macrophages served as a positive control for amastigote viability. After 48 h of treatment, the supernatant was collected, and the coverslips were stained with Panoptic. To determine the IC_50_, amastigotes were counted in 100 macrophages per coverslip, with the untreated control set as 100% intracellular amastigotes. The IC_50_ was then calculated using dose–response curve regression analysis.

### 3.12. Determination of NO

The supernatant from *L. infantum*-infected macrophages, treated as described in Section 3.11, was collected to assess NO production using the Griess reaction [71]. In short, 100 µL of the collected supernatants were transferred to 96-well microplates, followed by the addition of an equal volume of Griess reagent (0.5% sulfanilamide and 0.05% naphthylenediamine dihydrochloride in 5% phosphoric acid) to each well. The plates were then incubated at room temperature in the dark for 20 min. After incubation, the absorbance was measured at 570 nm. The nitrite concentrations were calculated using a sodium nitrite standard curve (0.195 to 100 µM).

### 3.13. Statistical Analysis

The in vitro biological assays were performed in triplicate and repeated three times independently. Data normality was assessed using the Shapiro–Wilk test, followed by a one-way ANOVA with Tukey’s *post-hoc* test for comparisons. Statistical analysis was conducted using GraphPad 8.0 software (GraphPad Software, Boston, MA, USA), with significance set at *p* < 0.05.

## Figures and Tables

**Table 1:** Half-maximum (IC_50_) values of *C. longa* extracts and curcumin (**1**) against *Li*ARG.

	*Li*ARG IC_50_ ± SE [µg/mL]
HEXCURC	14.4 ± 2.4 ^a^
DCCURC	10.04 ± 1.5 ^a^
ETOHCURC	52.64 ± 0.3 ^b^
Curcumin (**1**)	17.55 ± 0.4 ^a^
Quercetin (**7**) *	<6.25

Results are expressed as the mean ± standard error from three independent experiments. Means with different letters indicate significant differences (One-way ANOVA, Tukey post hoc test, *p* < 0.05). HEXCURC, *C. longa* hexane extract; DCCURC, *C. longa* dichloromethane extract; and ETOHCURC, *C. longa* ethanol extract. * **7** was used as reference inhibitor of *Li*ARG [7].

**Table 2:** In silico evaluation of pharmacokinetic and toxicological endpoints of the curcuminoids and turmerones.

Pharmacokinetic and Toxicological Endpoints	Compounds					
	1	2	3	4	5	6
Lipinski’s rule of 5	0	0	0	0	0	0
Unbound (%) to plasma protein	6.7	6.2	7.7	8.9	12.1	11.2
P-glycoprotein interaction	Yes	Yes	Yes	No	Yes	Yes
Mutagenicity (Ames test)	No	No	No	No	No	No
Carcinogenicity (rats)	No	No	No	No	No	No
Acute toxicity (rats)	No	No	No	No	No	No
Cardiotoxicity (hERG)	No	No	No	No	No	No
Hepatotoxicity	No	No	No	No	Yes	Yes

Curcumin (**1**); demethoxycurcumin (**2**); bisdemethoxycurcumin (**3**); ar-turmerone (**4**); α-turmerone (**5**), and β-turmerone (**6**).

**Table 3:** Cytotoxicity (CC_50_) against RAW 264.7 macrophages and VERO renal epithelial cells and hemolytic activity (CH_50_) against red blood cells (RBCs) of *C. longa* extracts and curcumin (**1**), expressed in μg/mL.

	RAW 264.7	VERO	RBC
HEXCURC	47.87 ± 6.9 ^a,b^	60 ± 3 ^a^	119.65 ± 3.4 ^a^
DCCURC	55.08 ± 5.4 ^a,b^	58 ± 3.7 ^a^	101.85 ± 9.6 ^a^
ETOHCURC	70.16 ± 3.3 ^a^	89.3 ± 7.9 ^a^	144.15 ± 13.35 ^a^
Curcumin (**1**)	69.75 ± 2.8 ^a^	n.d.	>200
PAT	30.8 ± 0.73 ^b^	23.9 ± 1.5 ^b^	>100

Results are expressed as the mean values ± standard error from three independent experiments. Means with different superscript letters within a column indicate significant differences (One-way ANOVA, Tukey post hoc test, *p* < 0.05). HEXCURC: *C. longa* hexane extract; DCCURC: *C. longa* dichloromethane extract; ETOHCURC: *C. longa* ethanol extract; PAT: potassium antimony(III) tartrate hydrate, as a source of trivalent antimony (Sb(III)), reference drug control; n.d.: not determined.

**Table 4:** Assessment of *C. longa* extracts and **1** anti-promastigotes activity and selectivity indexes (SI) based on macrophages (RAW 264.7) and parasite ratio (CC_50_/IC_50_).

	*L. amazonensis*		*L. infantum*	
	IC_50_ ± SE [µg/mL]	SI	IC_50_ ± SE [µg/mL]	SI
HEXCURC	102.3 ± 7.6 ^a^	0.47	69.6 ± 0.2 ^a^	0.7
DCCURC	59.6 ± 10.3 ^a,b^	0.9	28.2 ± 1.5 ^b^	1.95
ETOHCURC	53.3 ± 9.9 ^b,c^	1.3	74.7 ± 1.4 ^a^	0.94
Curcumin (**1**)	5.5 ± 0.45 ^d^	12.7	15.5 ± 1.7 ^c^	4.5
PAT	11.9 ± 3.1 ^c,d^	2.6	<6.25	>4.9

Results are expressed as the mean values ± standard error from three independent experiments. Means with different superscript letters within a column indicate significant differences (One-way ANOVA, Tukey post hoc test, *p* < 0.05). HEXCURC: *C. longa* hexane extract; DCCURC: *C. longa* dichloromethane extract; ETOHCURC: *C. longa* ethanol extract; PAT: potassium antimony(III) tartrate hydrate, as a source of trivalent antimony (Sb(III)), reference drug control.

## Data Availability

The original contributions presented in this study are included in the article and Supplementary Material. Further inquiries can be directed to the corresponding authors.

## Supplementary Materials

The following supporting information can be downloaded at: https://www.mdpi.com/article/10.3390/ph18060851/s1, Figure S1. Chromatographic profiles obtained through gas chromatography coupled with mass spectrometry (GC-MS); Figure S2. Effect of *C. longa* extracts, curcumin, and quercetin (reference inhibitor) on *Li*ARG activity; Figure S3. Overlay of re-docked ABH (blue) and co-crystallized ABH (green) after re-docking approach in *Lm*ARG; Figure S4. Cytotoxic effect of *C. longa* extracts, curcumin, and PAT (reference drug control) against different mammalian cells; Figure S5. Dose-response curves of *C. longa* extracts, curcumin, and PAT (reference drug control) against *L. amazonensis* and *L. infantum* promastigotes; Figure S6. Effect of curcumin and PAT (reference drug control) on intracellular *L. infantum* amastigote load.
