# Supplementary material for: In Vitro and In Silico Assessments of Curcuminoids and Turmerones from Curcuma longa as Novel Inhibitors of Leishmania infantum Arginase
Source: Pharmaceuticals (Basel). 2025 Jun 6;18(6):851. doi: 10.3390/ph18060851 (PMC12195615; doi:10.3390/ph18060851)
Supplement: Supplementary file 1 [file pharmaceuticals-18-00851-s001.zip › pharmaceuticals-3656663-supplementary.pdf]

pharmaceuticals

Supplementary Material

In Vitro and In Silico Assessments of Curcuminoids and Turmerones from *Curcuma longa* as Novel Inhibitors of *Leishmania infantum* Arginase

Flora F. S. Spíndola [1], Anderson S. Pinheiro [2,†], Maria Athana Mpalantinos [3], Jefferson R. A. Silva [4], Walter S. M. F. Neto [4], Raissa A. Conceição [1,5], Eduarda M. Barreto [5], Barbara A. Abrahim-Vieira [1,5], Carlos R. Rodrigues [1,5], Alessandra M. T. Souza [1,5], Dirlei Nico [6], Ana Claudia F. Amaral [3], Andreza R. Garcia [1,*] and Igor A. Rodrigues [1,7,*]

1 Programa de Pós-Graduação em Ciências Farmacêuticas, Faculdade de Farmácia, Universidade Federal do Rio de Janeiro, Rio de Janeiro 21941-902, RJ, Brazil; flora_fernanda@hotmail.com (F.F.S.S.); raissa.conceicao9@gmail.com (R.A.C.); babi_abrahim@hotmail.com (B.A.A.-V.); rangelfarmacia@gmail.com (C.R.R.); amtsouza2@yahoo.com.br (A.M.T.S.)
2 Departamento de Bioquímica, Instituto de Química, Universidade Federal do Rio de Janeiro, Rio de Janeiro 21941-902, RJ, Brazil; pinheiro@iq.ufrj.br
3 Laboratório de Produtos Naturais e Derivados, Farmanguinhos Fiocruz, Manguinhos, Rio de Janeiro 21041-250, RJ, Brazil; maria.mpalantinos@gmail.com (M.A.M.); aamaral_99@yahoo.com.br (A.C.F.A.)
4 Laboratório de Cromatografia, Departamento de Química, Instituto de Ciências Exatas, Universidade Federal do Amazonas, Manaus 69077-000, AM, Brazil; jrocha01@yahoo.com.br (J.R.A.S.); wssottoo@gmail.com (W.S.M.F.N.)
5 Departamento de Fármacos e Medicamentos, Faculdade de Farmácia, Universidade Federal do Rio de Janeiro, Rio de Janeiro 21941-902, RJ, Brazil; brrduda@gmail.com
6 Departamento de Microbiologia Geral, Instituto de Microbiologia Paulo de Góes, Universidade Federal do Rio de Janeiro, Rio de Janeiro 21941-902, RJ, Brazil; dirlei@micro.ufrj.br
7 Departamento de Produtos Naturais e Alimentos, Faculdade de Farmácia, Universidade Federal do Rio de Janeiro, Rio de Janeiro 21941-902, RJ, Brazil
* Correspondence: raposo.arg@gmail.com (A.R.G.); igor@farmacia.ufrj.br (I.A.R.)
† In memoriam.

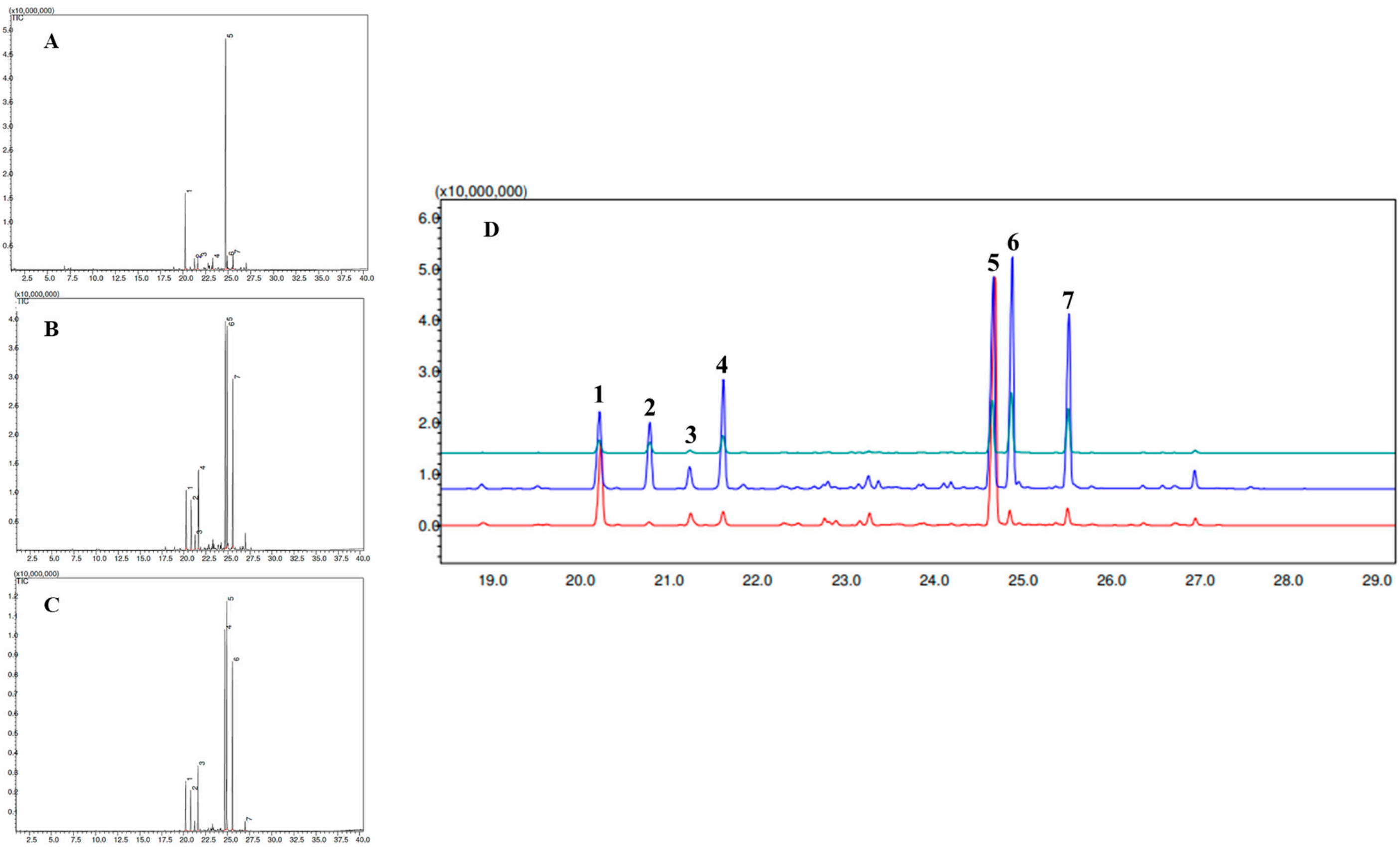

Figure S1. Chromatographic profiles obtained through gas chromatography coupled with mass spectrometry (GC-MS). (A) Total ion chromatogram (TIC) of the hexane extract (HEXCURC). (B) TIC of the dichloromethane extract (DCCURC). (C) TIC of the ethanol extract (ETOHCURC). (D) Partial ion current chromatogram of *Curcuma longa* extracts. Different colors represent different extracts: green for ETOHCURC, blue for DCCURC, and red for HEXCURC. The numbered peaks correspond to the following compounds: 1 – α-curcumene; 2 – zingiberene; 3 – β-bisabolene; 4 – β-sesquiphellandrene; 5 – β-turmerone; 6 – ar-turmerone; 7 – α-turmerone.

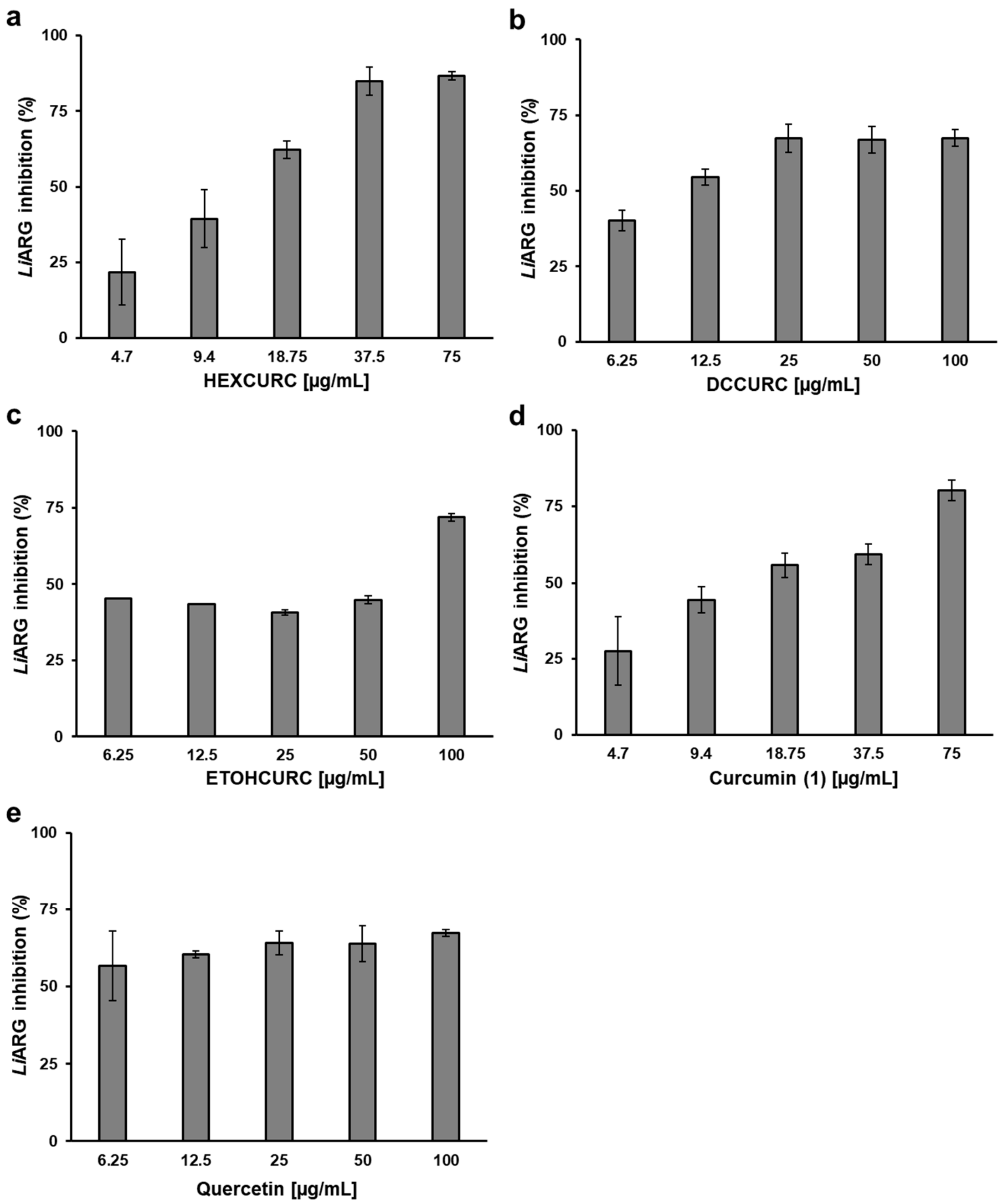

Figure S2. Effect of *C. longa* extracts, curcumin, and quercetin (reference inhibitor) on *Li*ARG activity. (a) HEXCURC, (b) DCCURC, (c) ETOHCURC, (d) curcumin, and (e) quercetin. Each bar represents the mean of three independent experiments performed in triplicate.

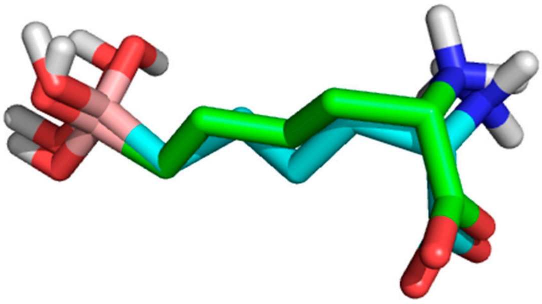

Figure S3. Overlay of re-docked ABH (blue) and co-crystallized ABH (green) after re-docking approach in *Lm*ARG. The best conformer identified in the re-docking analysis was the third out of four possible conformations of the lowest energy cluster, and the overlay with the crystallographic structure showed an RMSD of 0.79Å concerning the reference ligand (Figure S3). Therefore, the methodology was considered validated.

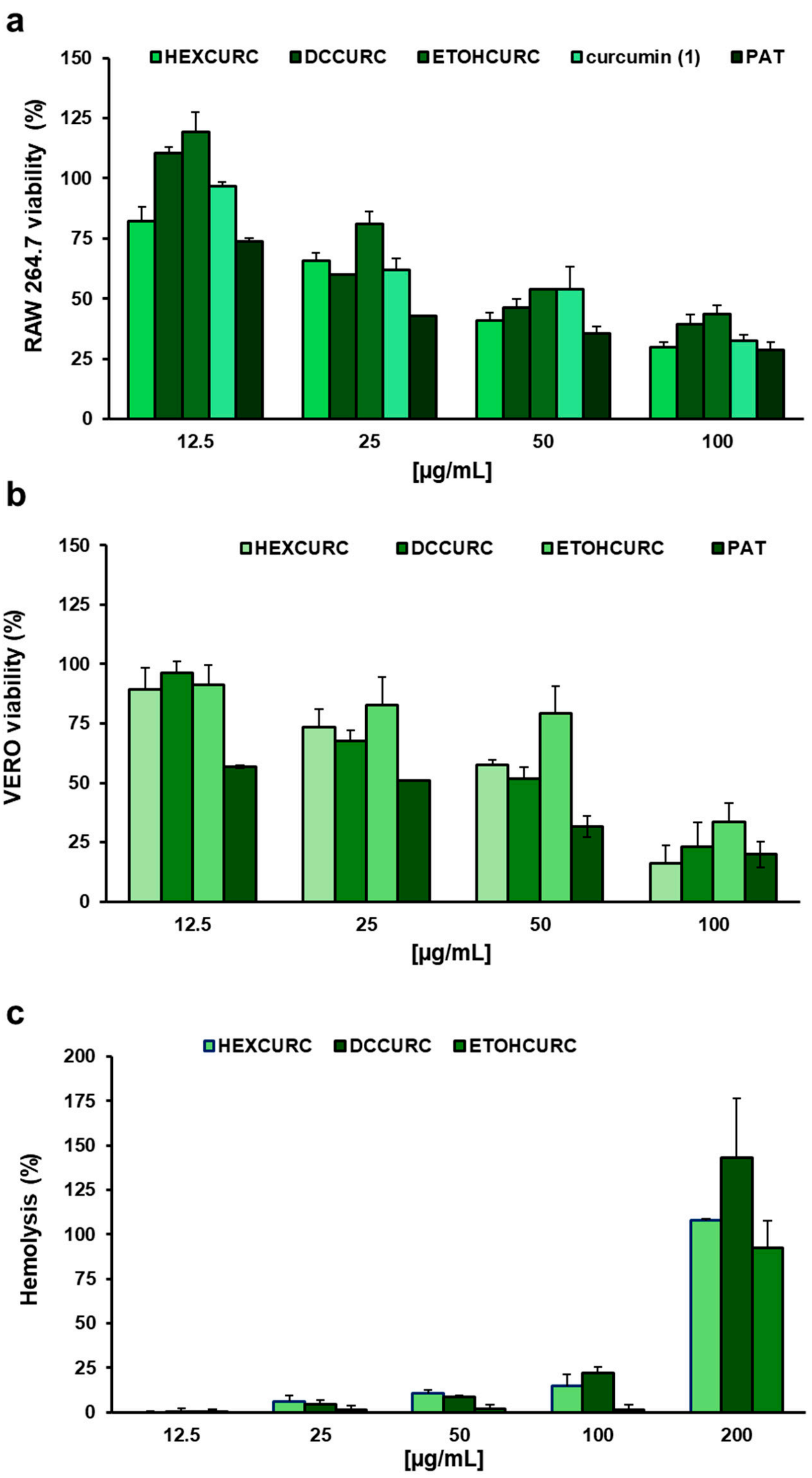

Figure S4. Cytotoxic effect of *C. longa* extracts, curcumin, and PAT (reference drug control) against different mammalian cells. (a) RAW 264.7 and (b) VERO cells, and (c) red blood cells. Each bar represents the mean of three independent experiments performed in triplicate.

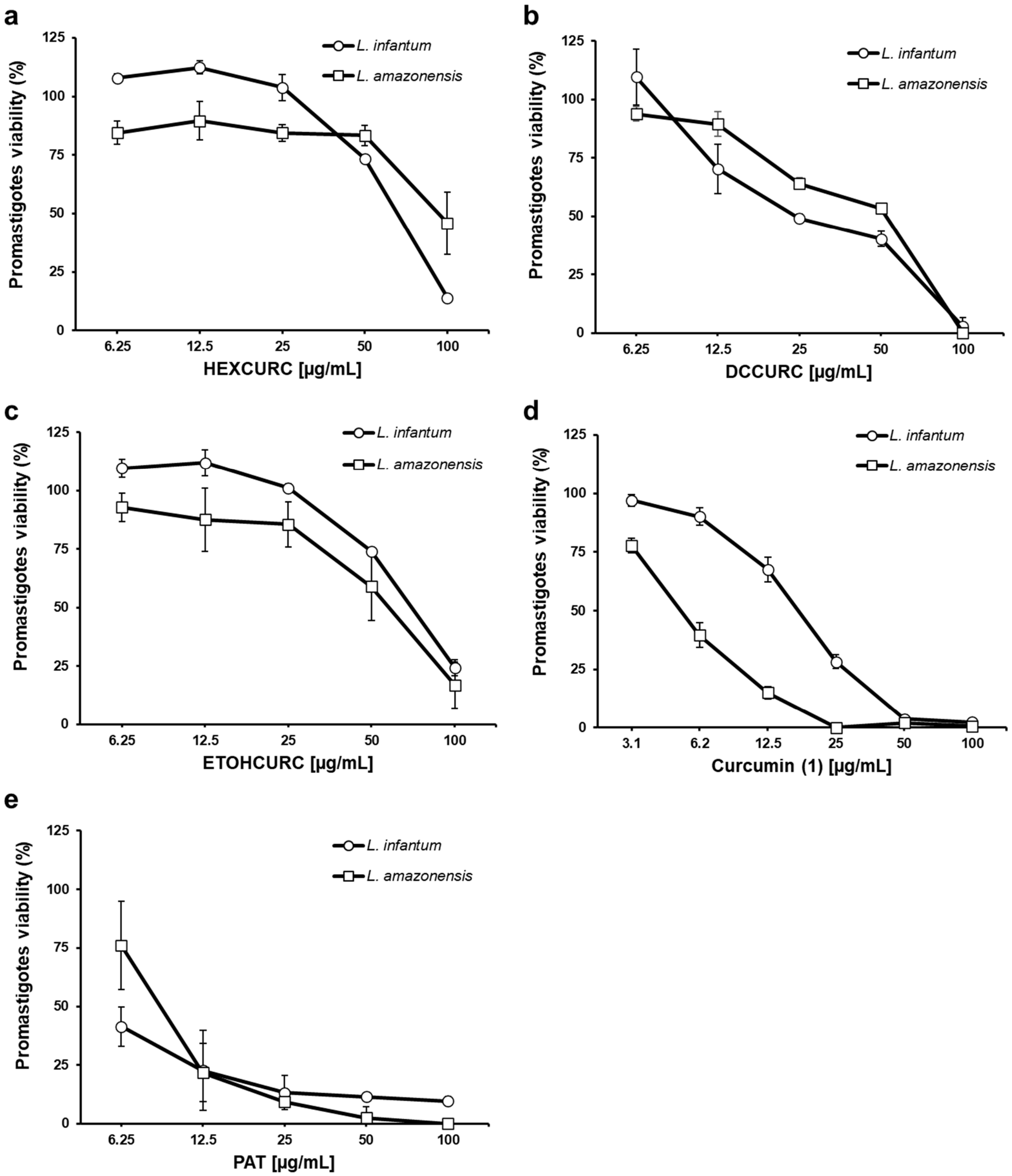

Figure S5. Dose-response curves of *C. longa* extracts, curcumin, and PAT (reference drug control) against *L. amazonensis* and *L. infantum* promastigotes. (a) HEXCURC, (b) DCCURC, (c) ETOHCURC, (d) curcumin, (e) PAT. Each point indicates the mean of three independent experiments performed in triplicate.

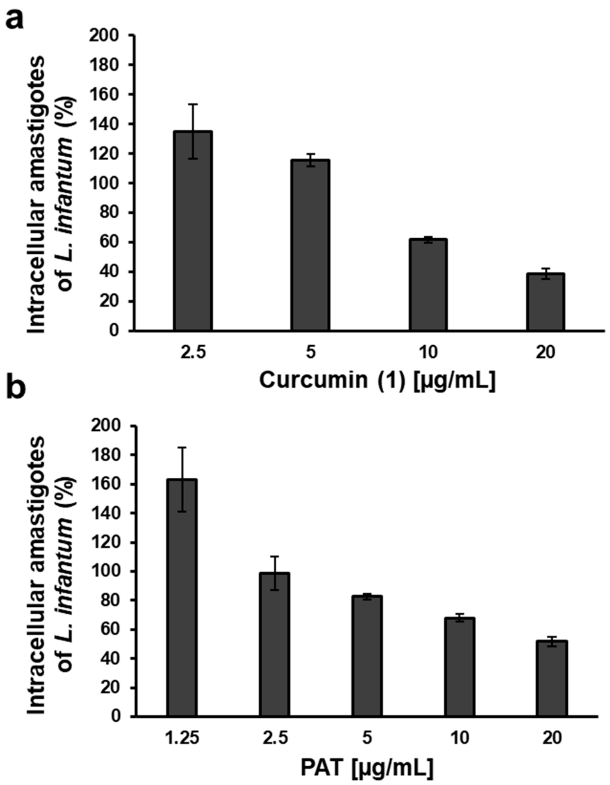

Figure S6. Effect of curcumin and PAT (reference drug control) on intracellular *L. infantum* amastigote load. (a) Infected RAW 264.7 macrophages treated with curcumin; (b) Infected RAW 264.7 macrophages treated with PAT. Each bar represents the mean of two experiments performed in duplicate.
